# A Randomized Controlled Trial of the Genistein Plus Bakuchiol and Vitamins (GEN^+^) Product for Male Facial Skin: Effects on Skin Appearance and Properties

**DOI:** 10.1111/srt.70310

**Published:** 2026-01-21

**Authors:** Mingkwan Na Takuathung, Kantirat Yaja, Jakkrit Aisara, Preeyaporn Klinjan, Pannita Anek, Ratchanon Inpan, Tattiya Kantasa, Jeeraporn Chitphan, Kankanit Yeerong, Supanimit Teekachunhatean, Nut Koonrungsesomboon

**Affiliations:** ^1^ Department of Pharmacology, Faculty of Medicine Chiang Mai University Chiang Mai Thailand; ^2^ Clinical Research Center for Food and Herbal Product Trials and Development (CR‐FAH), Faculty of Medicine Chiang Mai University Chiang Mai Thailand; ^3^ Office of Research Administration Chiang Mai University Chiang Mai Thailand

**Keywords:** bakuchiol, claim substantiation, genistein, male skincare, safety testing, skin physiology/structure

## Abstract

**Background:**

With increasing life expectancy, the aging population, particularly in Asia, is expanding rapidly. Combined with intense year‐round ultraviolet exposure, this accelerates skin aging. Male skin also exhibits distinct aging characteristics. This study aims to evaluate the efficacy and safety of the Genistein Plus Bakuchiol and Vitamins (GEN^+^) product on male facial skin.

**Materials and Methods:**

A randomized, double‐blind, placebo‐controlled trial was conducted in men aged 45–65 years. Participants were assigned to receive either the GEN^+^ or placebo (PLA) product in a 1:1 ratio and applied the product twice daily for 12 weeks. Facial skin assessments were performed at baseline and at weeks 4, 8, and 12. Evaluated parameters included skin color and consistency, spots, pigmentation, redness, acne, elasticity, hydration, barrier function, and wrinkles. Compliance and satisfaction were also monitored.

**Results:**

Eighty male participants were enrolled (GEN^+^: *n* = 40; PLA: *n* = 40). After 12 weeks, the GEN^+^ group demonstrated significant improvements in cheek skin lightening (MD = 0.55, 95% CI: 0.03 to 1.08, *p* = 0.04) and color consistency (MD = –0.56, 95% CI: –1.06 to –0.06, *p* = 0.03), along with a reduction in forehead spots (MD = –0.60, 95% CI: –1.15 to –0.06, *p* = 0.03). No significant differences were observed in melanin‐, erythema‐, or acne‐related parameters. Skin property parameters did not differ significantly between groups, except for skin roughness. Adverse events were mild and self‐resolving. While satisfaction scores did not differ significantly, the GEN^+^ group reported higher scores across most domains.

**Conclusion:**

The GEN^+^ product demonstrated promising improvements in male facial skin appearance, including skin lightening, color consistency, and spot reduction. The product was well‐tolerated and offered a safe, targeted skincare solution for men.

**Trial Registration:**

ClinicalTrials.gov identifier: TCTR20240312001

## 1 Introduction

Globally, life expectancy has increased, leading to a growing proportion of older individuals and significant demographic shifts [1]. Asia, home to half of the world's population, is experiencing particularly rapid population aging. By 2050‐2060, individuals aged 65 and older are projected to comprise 33.7% of the population in Eastern Asia and 20.3% in Southeast Asia [2, 3]. These trends underscore regional variations in aging and raise concerns about age‐related health issues, particularly skin aging. Skin aging can be classified as intrinsic aging or extrinsic aging [4]. The latter, also known as photoaging [4], is influenced by environmental factors prevalent in Asia. The issue is exacerbated by intense year‐round ultraviolet (UV) radiation in many parts of Asia, particularly subtropical and tropical regions such as Southeast Asia, where UV indices are often classified as “very high” to “extreme” [5, 6]. Prolonged UV exposure accelerates skin aging, posing a growing public health challenge [7]. Studies on Asian populations frequently report pigmentary changes as a major component of photoaging [8], including conditions such as actinic or senile lentigo, post‐inflammatory hyperpigmentation (PIH), melasma, and vitiligo, all of which significantly impact quality of life [8, 9, 10].

While aging affects all individuals, evidence suggests that male skin exhibits distinct aging characteristics compared to female skin. Intrinsic factors influencing skin aging include genetic factors and hormonal changes that naturally occur with age [11]. Male skin, driven by higher sebaceous gland activity under the influence of sex hormones, produces more sebum, leading to differences in skin texture and function [11]. These hormonal factors not only contribute to thicker skin and greater pigmentation in men but also make them more prone to deeper facial wrinkles and pronounced sagging, particularly in the lower eyelid region [11, 12]. Extrinsic factors, such as environmental pollution and UV radiation, further accelerate the skin aging process by promoting oxidative stress and the production of reactive oxygen species (ROS), which damage skin cells [13]. With advancing age, men experience a decline in antioxidant defenses, leaving their skin more susceptible to damage [14]. Studies show that men also have reduced innate antioxidant defenses and are more vulnerable to UV‐induced immunosuppression compared to women [15, 16]. Lifestyle factors exacerbate these effects; for example, frequent shaving can irritate the skin, causing dryness, redness, and clogged pores, which may result in conditions like razor bumps and acne [17]. Furthermore, men are more likely to work in outdoor environments, leading to prolonged sun exposure, and they tend to adopt fewer sun‐protective behaviors, such as regular sunscreen use [18]. These combined factors contribute to rougher skin texture, deeper wrinkles, uneven pigmentation, and an overall dull appearance, highlighting the significance of skin aging as a concern for men.

In response to these challenges, the organic skincare market has seen rapid growth. Herbal cosmetics derived from natural ingredients are increasingly popular due to their safety, suitability for all skin types, affordability, minimal adverse effects, and environmentally friendly nature [19]. The growing interest in organic lifestyles has fueled the exploration of natural compounds as safe and effective alternatives for addressing skin aging. Phytoestrogens, plant‐based compounds that mimic estrogen activity in the human body, have gained attention for their non‐hormonal skin benefits [20]. Estrogen receptor beta (ERβ) is present in both male and female skin, playing a crucial role in maintaining skin integrity through its expression in dermal fibroblasts and epidermal keratinocytes [21, 22]. Genistein, a biologically active isoflavone and potent phytoestrogen found primarily in soybeans, exerts its effects through estrogen receptor beta (ERβ) activation [23]. Research indicates that isoflavones (including genistein) enhances collagen and elastin production [24], reduces oxidative stress [25], and improves skin hydration and photoaging‐related concerns [26]. Additionally, its anti‐inflammatory properties help alleviate irritation and maintain skin balance [27]. Another noteworthy natural compound, bakuchiol, primarily derived from the seeds of *Psoralea corylifolia*, has gained attention for addressing male‐specific skin concerns. Often referred to as a “functional analogue of retinol [28],” bakuchiol has demonstrated comparable efficacy to over‐the‐counter vitamin A derivatives but with fewer adverse effects. Its antioxidant, anti‐inflammatory, and anti‐aging properties make it a promising ingredient for combating skin aging [28, 29]. When combined with other well‐researched ingredients, these compounds enhance skin health. Ascorbic acid (Vitamin C) and tocopherol (Vitamin E) are potent antioxidants that protect against oxidative stress and support collagen synthesis, contributing to skin elasticity and firmness [30, 31, 32]. Niacinamide (Vitamin B3) strengthens the skin barrier and improves hydration and resilience [33]. Ceramides further reinforce the barrier function, prevent moisture loss, and help maintain overall skin hydration [34].

Despite these promising findings, research specifically examining the effects of isoflavones and bakuchiol on male skin remains limited. Building on the known benefits of these ingredients, we propose a novel formulation, the Genistein Plus Bakuchiol and Vitamins (GEN^+^) product. This formulation combines genistein, bakuchiol, key vitamins (Vitamin C, Vitamin E, and Vitamin B3), and ceramides. By leveraging the synergistic effects of these components, GEN^+^ aims to support overall skin health and address the unique concerns associated with male skin aging, focusing on parameters such as skin color and color consistency, spots, pigmentation, redness, acne, elasticity, hydration, skin barrier function, and wrinkle appearance. Additionally, the study assessed male participants’ satisfaction with their skin and the product, with all findings contributing to the expanding body of knowledge in male skin care research.

## 2 Material and Methods

### 2.1 Study Design and Setting

This randomized, double‐blind, placebo‐controlled trial with a two‐arm, parallel‐group design was conducted at the Clinical Research Center for Food and Herbal Product Trials and Development (CR‐FAH) and the Department of Pharmacology, Faculty of Medicine, Chiang Mai University, Thailand, from June to September 2024. The trial was registered prospectively with the Thai Clinical Trials Registry before participant enrollment. Ethical approval was obtained from the Research Ethics Committee of the Faculty of Medicine, Chiang Mai University (No. 020/2024). The study complied with the Declaration of Helsinki (2013) and Good Clinical Practice guidelines (ICH E6(R2)). All participants provided written informed consent before joining the study.

### 2.2 Study Participants and Sample Size Determination

The study recruited men aged 45–65 years with a body mass index (BMI) of 18–30 kg/m^2^ who were able to read and write in Thai. Participants were excluded if they had received estrogen hormone or isoflavone treatment within six months before screening. Additional exclusion criteria included any skin conditions (e.g., periorificial dermatitis, skin cancer, or infections) or factors interfering with skin assessment, such as cuts, sunburn, birthmarks, tattoos, extensive scarring, excessive hair growth, or acne. Participants who had used topical anti‐wrinkle treatments or undergone aesthetic procedures within 30 days prior to screening or those with hypersensitivity to soy products or any other ingredients in the study products were also excluded. Individuals with a history of narcotic or psychotropic substance use, excessive alcohol consumption, or regular smoking were ineligible.

This trial was planned to enrol 80 participants. The sample size was calculated for testing two independent means (two‐tailed test) based on a prior study [35]. Assuming a mean change in skin roughness of –10, a standard deviation of 14, a significance level (*α*) of 0.05, and a statistical power of 80%, an effect size of 0.72 was used to determine that 32 participants per group were required. Accounting for an anticipated 20% dropout rate, the target enrollment was set at 40 participants per group, totaling 80 participants.

### 2.3 Study Intervention and Comparator

The investigational products, including Genistein Plus Bakuchiol and Vitamins (GEN^+^) product and placebo (PLA) product, were manufactured by KK Cosmetic Institute, Chiang Mai, Thailand. The GEN^+^ cream contained 4% genistein along with additional ingredients, including bakuchiol, key vitamins (Vitamin C, Vitamin E, and Vitamin B3), and ceramides. The placebo product was formulated with a non‐active cream base and included coloring agents and fragrances to match the appearance of the GEN^+^ product.

### 2.4 Randomization, Allocation Concealment, and Blinding

Prior to the trial, computer‐generated randomization was conducted using a 1:1 allocation ratio with a block size of 10. Research staff not involved in the investigational aspects of the study dispensed either the GEN^+^ or PLA product to participants. The allocation sequence was concealed from the investigators using sequentially numbered, opaque, sealed envelopes, which were opened only after participant eligibility was confirmed. The investigator team, including those assessing clinical outcomes, remained blind to group assignments throughout the study. Both the GEN^+^ and PLA products were provided in identical opaque polypropylene vacuum pump plastic bottles.

### 2.5 Study Procedures and Outcome Assessment

Participants were screened for eligibility before the trial. An occlusive patch test was conducted to assess potential allergic reactions. Approximately 0.2 g or 0.2 mL of the GEN^+^ product was applied to a 3 × 3 cm^2^ area of intact skin on the back and covered with a waterproof occlusive patch for 48 hours. Participants were monitored for adverse events, such as allergic rashes. If allergic reactions occurred, participants were withdrawn from the study for safety reasons, and new volunteers were recruited as replacements.

At baseline, participants underwent an assessment of their facial skin condition. Following this, they were given a randomized product (GEN^+^ or PLA) and instructed to apply ∼0.3 mL (approximately two pumps) of the product twice daily to their facial skin for 12 weeks. During the trial, participants were allowed to follow their usual makeup, cleansing, and sunscreen routine; however, they were instructed to avoid using other cosmetic or nourishing products and were prohibited from consuming herbs or nutritional supplements that could influence skin condition.

Facial skin assessments were conducted at baseline and at the end of Weeks 4, 8, and 12. At each follow‐up, participants cleansed their skin with water and then waited 20 minutes to ensure standardized skin conditions before undergoing assessments. Measurements were taken from both the left and right sides of the face, specifically at the cheek and crow's feet areas. Each side was measured, and the average value was calculated to obtain a single representative value.

The study assessed eight domains of skin parameters to comprehensively evaluate facial skin outcomes. These included skin appearance domains (Facial Skin Color and Spot Parameters, Skin Color Parameters, Melanin and Erythema Parameters, and Acne Parameters) and skin properties domains (Elasticity Parameters, Hydration Parameters, Transepidermal Water Loss (TEWL) Parameters, and Wrinkle Parameters).

1. **Facial skin color and spot parameters** were measured using the Visioface 1000 D (Courage + Khazaka electronic GmbH, Cologne, Germany). The assessed variables included facial skin color (ΔL: change in lightness, and ΔE: change in overall color difference) and spot parameters (spot count and area). Measurements were captured from a single picture of the entire face taken during each follow‐up visit. The analysis was performed by cropping specific locations, including the forehead, chin, and cheeks. For facial skin color analysis, two images were captured at each specific location (forehead, chin, and cheeks), resulting in two measurements per location: ΔL1 and ΔL2 for lightness changes and ΔE1 and ΔE2 for overall color difference.
2. **Skin color parameters** were measured using the Skin‐Colorimeter CL 400 (Courage + Khazaka electronic GmbH, Cologne, Germany). Variables included lightness (Avg. L), redness (Avg. A), pigmentation (Avg. B), and individual typology angle (Avg. ITA). Measurements were taken five times at random locations across the forehead, chin, cheeks, and nose. The average value for each location was calculated and used for analysis.
3. **Melanin and erythema parameters** were assessed using the Mexameter MX 18 (Courage + Khazaka electronic GmbH, Cologne, Germany). Measurements were taken randomly from the forehead, chin, cheek, and nose areas. The average of five repeated measurements was calculated.
4. **Acne parameters,** including porphyrin size, quantity (count), and intensity, were measured using the Visiopor PP34 (Courage + Khazaka electronic GmbH, Cologne, Germany). Measurements were taken three times at the forehead, chin, and cheeks, and the averages were calculated to represent each location.
5. **Elasticity parameters** were measured using the Cutometer dual MPA 580 (Courage + Khazaka electronic GmbH, Cologne, Germany). Measurements were taken three times on the forehead, chin, and cheek areas. The following parameters, commonly used to assess skin elasticity, were evaluated:
  - R0: Amplitude at the end of the suction phase
  - R2: Elasticity to total deformation ratio
  - R5: Net elastic ratio
  - R7: Immediate elastic recovery ratio
6. **Hydration parameters** were evaluated using the Corneometer CM 825 (Courage + Khazaka electronic GmbH, Cologne, Germany). Five random measurements were taken from the forehead, chin, and cheek areas, and the average value was calculated.
7. **Transepidermal water loss (TEWL) parameter** was measured using a Tewameter (Courage + Khazaka electronic GmbH, Cologne, Germany). For each assessment, three measurements were taken at random locations within the forehead, chin, and cheek areas. The average of these measurements was calculated to obtain a representative TEWL value for each location.
8. **Wrinkle parameters** were assessed through skin roughness measurements using the Skin Visioscan VC20 (Courage + Khazaka electronic GmbH, Cologne, Germany). These parameters included skin roughness depth (R1), maximum roughness (R2), average roughness (R3), smooth depth (R4), and arithmetic average roughness (R5). Measurements were taken at multiple facial locations, including the forehead, chin, cheek, and crow's feet areas. At each location, three measurements were performed, and the average value was calculated.

Moreover, participant satisfaction was assessed in terms of both the product and skin outcomes. Product satisfaction was evaluated based on the appearance, smell, color, texture, and toughness of the product. Skin satisfaction was assessed regarding perceived improvements in skin appearance, such as brightness, health, pore size, and reduction in redness. A 10‐point questionnaire was used at each follow‐up visit, with higher scores indicating greater satisfaction. Any adverse events, whether observed by investigators or self‐reported by participants, were recorded throughout the study. At each follow‐up visit, participants were required to bring their product‐usage diary and return any remaining study products. Product compliance was determined by the remaining weight of the cream in the vial. Participants who used less than 50% of the prescribed dosage were considered poorly compliant.

### 2.6 Statistical Analysis

Statistical analyses were conducted using R‐Studio (version 2023.06.1; R Foundation, Vienna, Austria, build 524). Both the intention‐to‐treat (ITT) and per‐protocol (PP) approaches were employed to evaluate study outcomes. The ITT analysis included all randomly assigned participants, analyzed based on their initial group allocation. The PP analysis excluded participants with poor compliance.

Demographic and outcome parameters were presented as mean ± standard deviation (SD) and mean differences (MD) with 95% confidence intervals (CI). Between‐group differences were analyzed using the Student's *t*‐test for the satisfaction questionnaires, employing the “stats” package (version 4.4.0). Within‐group differences in skin outcomes at different follow‐up time points were evaluated using repeated measures ANOVA, implemented via the “rstatix” package (version 0.7.2). If significant differences were observed, post‐hoc analyses with Bonferroni adjustments were performed.

Analysis of covariance (ANCOVA) was used to control potential confounding variables, such as baseline characteristics and individual compliance rates, using the “car” package version 3.1–3. Regression analysis was also conducted to assess the impact of compliance rates on each parameter using the “stats” package (version 4.4.0). All statistical tests were two‐sided, with a significance threshold set at *p* < 0.05.

## 3 Results

**FIGURE 1:**
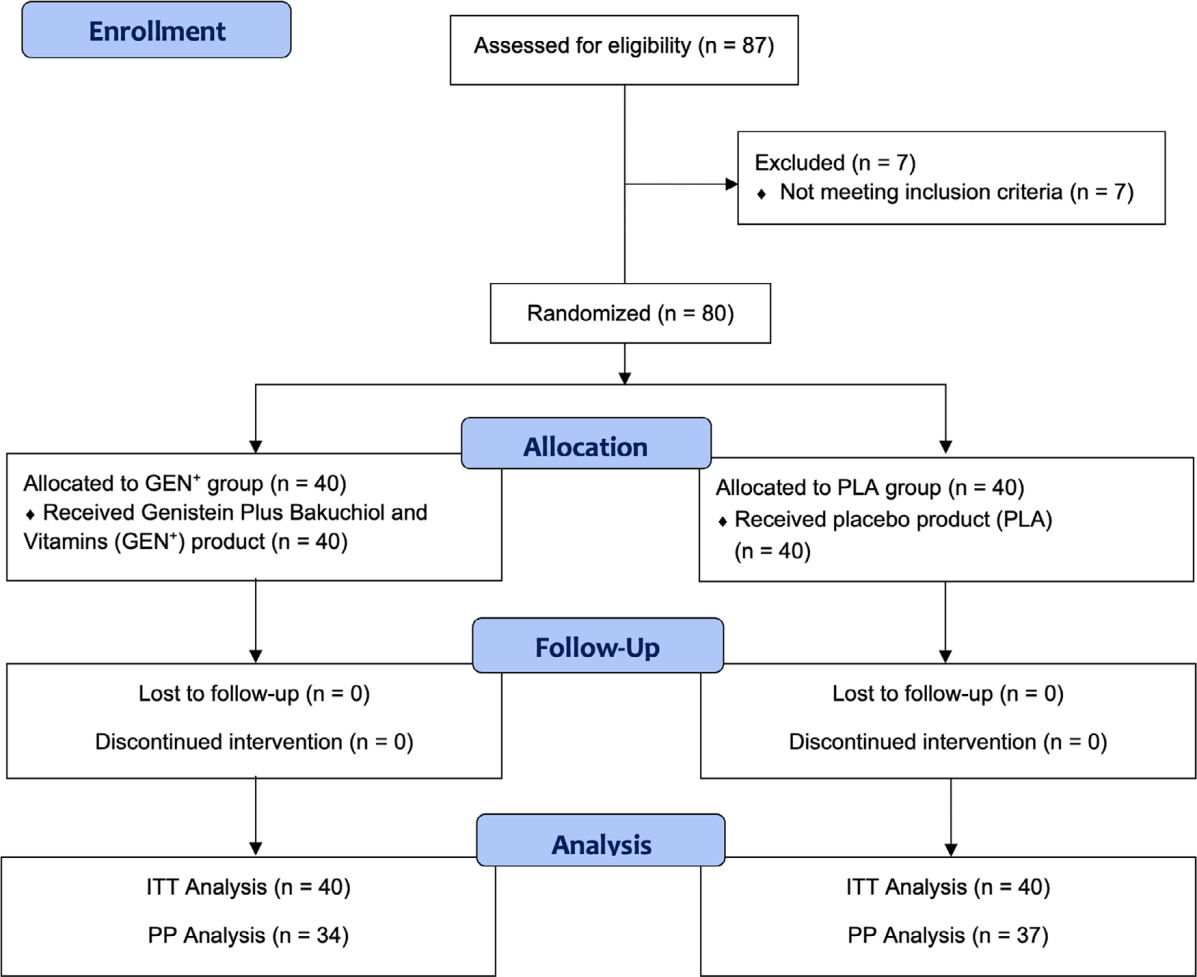
Consort flow diagram.

A total of 87 men were initially screened for eligibility. Seven participants were excluded due to exceeding the BMI range (*n* = 2), undergoing surgery on the facial area (*n* = 2), receiving botulinum toxin treatments on the facial area (*n* = 1), heavy smoking (*n* = 1), and being younger than the inclusion criteria (*n* = 1). Finally, 80 male participants were enrolled and randomly assigned to receive either the GEN^+^ product or the PLA product (Figure 1).

The participants had an age range from 45 to 64 years. The mean ages of participants in the GEN^+^ and PLA groups were 52.02 ± 5.82 years and 52.85 ± 6.01 years, respectively. The BMI values in the GEN^+^ and PLA groups were 24.31 ± 2.71 and 23.83 ± 2.92, respectively. The baseline skin characteristics of the participants are presented in Table S1.

None of the participants were lost to follow‐up or withdrew during the study period. However, nine individuals (six in the GEN^+^ group and three in the PLA group) used the product less than 50% of the prescribed dosage and were classified as poorly compliant. Consequently, the ITT analysis included all 80 participants (40 in the GEN^+^ group and 40 in the PLA group), while the PP analysis included 71 participants (34 in the GEN^+^ group and 37 in the PLA group) (Figure 1).

Due to differences in baseline skin characteristics and variations in compliance in this study, the findings were derived from ANCOVA analysis, adjusted for baseline values and compliance rates. After evaluating facial skin parameters following a 12‐week intervention focusing on visual and measurable surface characteristics, the GEN^+^ group demonstrated greater improvements compared to the PLA group in some aspects.

### 3.1 Skin Appearance

**FIGURE 2:**
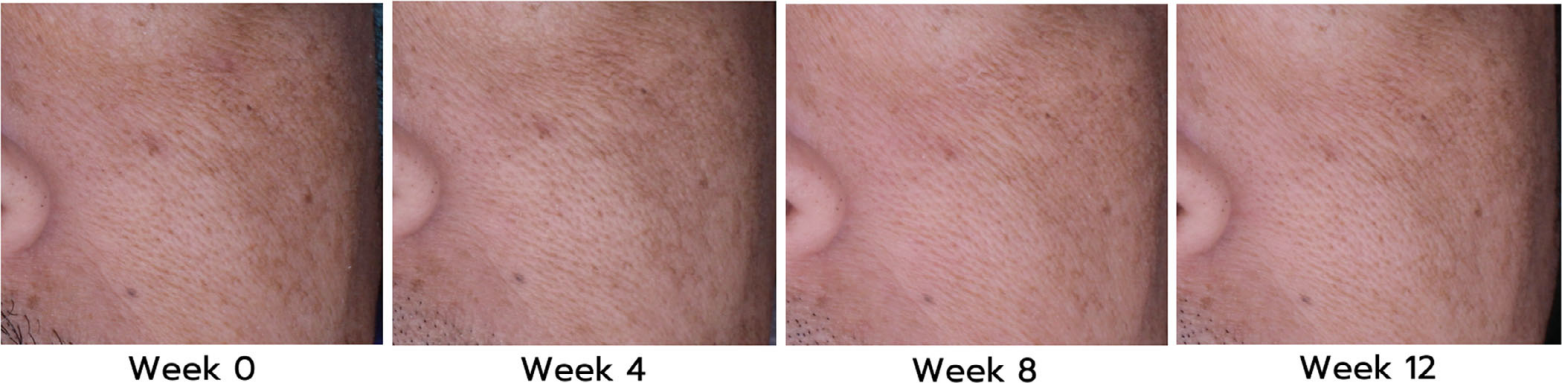
Visioface images of the cheek area showing changes in facial lightness (ΔL) and color consistency (ΔE) in the GEN^+^ group at baseline (Week 0) and Weeks 4, 8, and 12 following GEN^+^ product application.

Facial brightness, as indicated by the ΔL2 parameter, showed a higher trend in the GEN^+^ group compared to the PLA group, as analyzed by both the ITT and PP approaches. A statistically significant difference was observed in the cheek area for the GEN^+^ group when analyzed using either the ITT or PP approach (ITT: MD = 0.55, 95% CI: 0.03 to1.08, *p* = 0.04; PP: MD = 0.70, 95% CI: 0.17 to1.23, *p* = 0.01) (Figure 2). Additionally, the ΔE1 parameter, reflecting color differences, revealed a decreasing trend in the cheek area for the GEN^+^ group, with a statistically significant difference observed with PP analysis (ITT: MD = –0.39, 95% CI: –0.89 to 0.12, *p* = 0.13; PP: MD = –0.56, 95% CI: –1.06 to –0.06, *p* = 0.03), suggesting improved color consistency (Figure 2). The GEN^+^ group also exhibited a significant reduction in spot count compared to the PLA group in the forehead area in both the ITT and PP analyses (ITT: MD = –0.60, 95% CI: –1.15 to –0.06, *p* = 0.03; PP: MD = –0.61, 95% CI: –1.22 to –0.00, *p* = 0.04). No significant differences were observed between the two groups for other parameters, including melanin, erythema (skin redness), and acne‐related parameters (Porphyrins size (%), count, and intensity) (Table 1).

**TABLE 1:** Changes in facial skin appearance parameters after the 12‐week intervention between the GEN^+^ and PLA groups, including skin color, spot count, melanin, erythema, and porphyrins. The analysis comprised an intention‐to‐treat (ITT) analysis (*n* = 80; GEN^+^ = 40, PLA = 40) and a per‐protocol (PP) analysis of participants with compliance greater than 50% of the prescribed dosage (*n* = 71; GEN^+^ = 34, PLA = 37). The results were reported as mean differences (MD) with 95% confidence intervals (CI).

Locations	Forehead		Chin		Cheek (average left and right side)		Nose	
Parameters	ITT	PP	ITT	PP	ITT	PP	ITT	PP
**Facial skin color and spot parameters**								
ΔL1	0.33 [–0.23 to 0.89]	0.37 [–0.23 to 0.97]	0.30 [–0.44 to 1.04]	−0.09 [–0.84 to 0.67]	0.35 [–0.17 to 0.87]	0.40 [–0.16 to 0.96]		
ΔL2	0.39 [–0.13 to 0.92]	0.40 [–0.18 to 0.98]	0.66 [–0.19 to 1.52]	0.28 [–0.56 to 1.12]	0.55 [0.03 to 1.08]^a^	0.70 [0.17 to 1.23]^a^		
ΔE1	0.03 [–0.61 to 0.67]	0.00 [–0.72 to 0.72]	−0.43 [–1.04 to 0.19]	−0.05 [–0.71 to 0.61]	−0.39 [–0.89 to 0.12]	−0.56 [–1.06 to –0.06]^a^		
ΔE2	−0.06 [–0.53 to 0.40]	−0.10 [–0.61 to 0.40]	−0.24 [–0.97 to 0.50]	0.16 [–0.44 to 0.77]	−0.12 [–0.62 to 0.39]	0.00 [–0.52 to 0.53]		
Spot (count)	−0.60 [–1.15 to –0.06]^a^	−0.61 [–1.22 to –0.00]^a^	0.78 [–0.02 to 1.58]	−0.22 [–0.92 to 0.49]	0.46 [–0.15 to 1.07]	0.39 [–0.29 to 1.06]		
Spot area (px)	−360.59 [–832.06 to 110.89]	−379.06 [–904.45 to 146.33]	868.44 [–142.12 to 1879.01]	−230.88 [–933.46 to 471.71]	393.52 [–327.82 to 1114.86]	375.45 [–379.59 to 1130.49]		
**Skin color parameters**								
Avg L (arb. unit)	0.06 [–0.45 to 0.58]	0.05 [–0.50 to 0.60]	0.02 [–0.48 to 0.52]	0.12 [–0.44 to 0.68]	0.09 [–0.42 to 0.59]	0.12 [–0.43 to 0.67]	−0.14 [–0.85 to 0.58]	−0.34 [–1.08 to 0.40]
Avg A (arb. unit)	0.04 [–0.39 to 0.46]	−0.03 [–0.49 to 0.43]	0.07 [–0.35 to 0.50]	0.13 [–0.32 to 0.59]	0.05 [–0.29 to 0.40]	−0.03 [–0.41 to 0.36]	0.03 [–0.51 to 0.57]	−0.09 [–0.68 to 0.49]
Avg B (arb. unit)	0.12 [–0.31 to 0.54]	0.10 [–0.37 to 0.57]	−0.13 [–0.52 to 0.26]	−0.12 [–0.54 to 0.30]	0.25 [–0.14 to 0.65]	0.22 [–0.23 to 0.68]	−0.04 [–0.50 to 0.42]	0.09 [–0.41 to 0.60]
Avg. ITA (arb. unit)	0.25 [–1.79 to 2.29]	0.30 [–1.92 to 2.52]	−0.27 [–2.32 to 1.77]	0.22 [–2.08 to 2.51]	0.49 [–1.62 to 2.60]	0.80 [–1.51 to 3.12]	−0.42 [–2.97 to 2.14]	−1.30 [–3.95 to 1.35]
**Melanin and erythema parameters**								
Melanin	−1.41 [–12.74 to 9.92]	−1.08 [–13.72 to 11.57]	1.41 [–9.11 to 11.93]	0.68 [–10.85 to 12.20]	−4.85 [–15.33 to 5.62]	−5.56 [–17.16 to 6.05]	−6.45 [–21.21 to 8.32]	−2.96 [–18.65 to 12.73]
Erythema	−2.09 [–14.57 to 10.39]	−3.16 [–16.95 to 10.63]	−5.13 [–18.08 to 7.82]	−5.28 [–19.18 to 8.63]	5.77 [–3.80 to 15.35]	4.26 [–6.48 to 14.99]	−2.11 [–19.01 to 14.79]	−3.14 [–21.60 to 15.33]
**Porphyrins parameters**								
Size (%)	−0.05 [–0.32 to 0.22]	−0.09 [–0.39 to 0.21]	0.14 [–0.22 to 0.49]	0.20 [–0.18 to 0.58]	0.06 [–0.13 to 0.26]	0.07 [–0.12 to 0.27]		
Count	−1.82 [–4.92 to 1.28]	−1.74 [–5.25 to 1.76]	1.03 [–3.18 to 5.24]	0.88 [–3.31 to 5.06]	0.37 [–2.36 to 3.09]	−0.24 [–3.03 to 2.55]		
Avg. Intensity	−13.64 [–32.83 to 5.55]	−8.84 [–28.46 to 10.78]	0.51 [–6.22 to 7.23]	0.15 [–6.38 to 6.68]	−3.88 [–16.55 to 8.79]	−7.41 [–20.25 to 5.43]		

Abbreviations: arb. unit, arbitrary unit; Avg. A, average redness; Avg. B, average pigmentation; Avg. ITA, average individual typology angle; Avg. L, average lightness; Avg., average; px, pixels; ΔE, overall color difference; ΔL, change in lightness.

^a^ Statistically significant differences between groups (*p* < 0.05) when analyzed using ANCOVA, adjusted for baseline values and compliance rate.

The GEN^+^ group demonstrated improvements in facial brightness over time. Repeated measures ANOVA revealed significant group‐by‐time interactions, specifically in the cheek area, with an increase in the ΔL2 parameter and a decrease in the ΔE1 parameter. The ΔL2 values were statistically higher at Weeks 4, 8, and 12 compared to baseline, indicating consistent improvement in lightness. Moreover, the ΔE1 parameter significantly decreased at Weeks 4, 8, and 12 compared to the baseline, suggesting enhanced color uniformity over time. Furthermore, the reduction in spot count in the GEN^+^ group became statistically significant from Week 8 onward compared to the PLA group (Table S2).

### 3.2 Skin Properties

In terms of facial skin properties, including elasticity, hydration, and transepidermal water loss (TEWL), no significant differences were observed between the GEN^+^ and PLA groups across all parameters, whether analyzed by the ITT or PP approach. Exceptions were observed in wrinkle parameters assessed through skin roughness measurements, specifically R1 in the cheek areas (ITT: MD = 3.31, 95% CI: 0.55 to6.08, *p* = 0.02; PP: MD = 3.22, 95% CI: 0.18 to6.25, *p* = 0.04), R2 at the cheeks (ITT: MD = 2.78, 95% CI: 0.11 to5.46, *p* = 0.04; PP: MD = 2.56, 95% CI: –0.33 to 5.46, *p* = 0.08), R3 at the forehead (ITT: MD = 2.93, 95% CI: 0.03 to5.83, *p* = 0.047; PP: MD = 3.51, 95% CI: 0.31 to6.72, *p* = 0.03), and R3 at the cheeks (ITT: MD = 2.71, 95% CI: 0.72 to4.69, *p* = 0.008; PP: MD = 2.51, 95% CI: 0.35 to4.68, *p* = 0.02) (Table 2).

**TABLE 2:** Changes in facial skin properties parameters after the 12‐week intervention between the GEN^+^ and PLA groups, including elasticity, hydration, transepidermal water loss, and wrinkles. The analysis comprised an intention‐to‐treat (ITT) analysis (*n* = 80; GEN^+^ = 40, PLA = 40) and a per‐protocol (PP) analysis of participants with compliance greater than 50% of the prescribed dosage (*n* = 71; GEN^+^ = 34, PLA = 37). The results were reported as mean differences (MD) with 95% confidence intervals (CI).

Locations	Forehead		Chin		Cheek (average left and right side)		Crow's feet (average left and right side)	
Parameters	ITT	PP	ITT	PP	ITT	PP	ITT	PP
**Skin elasticity parameters**								
R0	0.01 [–0.03 to 0.06]	0.02 [–0.03 to 0.07]	−0.02 [–0.05 to 0.01]	−0.02 [–0.05 to 0.01]	0.03 [–0.00 to 0.05]	0.02 [–0.00 to 0.05]		
R2	−2.99 [–7.03 to 1.04]	−2.22 [–6.74 to 2.29]	1.21 [–2.54 to 4.96]	0.78 [–3.40 to 4.95]	−0.73 [–3.82 to 2.35]	−0.95 [–4.35 to 2.45]		
R5	−3.98 [–8.03 to 0.06]	−2.58 [–6.80 to 1.63]	1.33 [–2.57 to 5.23]	1.07 [–3.25 to 5.39]	−0.95 [–3.97 to 2.08]	−1.36 [–4.59 to 1.87]		
R7	−2.39 [–4.87 to 0.09]	−1.32 [–3.91 to 1.28]	0.49 [–2.14 to 3.12]	0.27 [–2.63 to 3.17]	−0.78 [–2.73 to 1.16]	−0.93 [–2.99 to 1.13]		
**Skin hydration parameters**								
Hydration value (arb. unit)	0.77 [–3.33 to 4.87]	1.95 [–2.28 to 6.18]	1.53 [–2.14 to 5.19]	2.42 [–1.48 to 6.32]	2.59 [–1.09 to 6.27]	1.88 [–2.05 to 5.82]		
**TEWL parameters**								
TEWL	−0.26 [–2.32 to 1.79]	−0.78 [–2.96 to 1.41]	−2.70 [–5.80 to 0.39]	−3.12 [–6.68 to 0.43]	−1.90 [–4.57 to 0.77]	−2.14 [–4.99 to 0.70]		
**Skin wrinkle parameters**								
R1 (arb. unit)	3.42 [–0.40 to 7.24]	4.11 [–0.13 to 8.36]	2.31 [–1.18 to 5.80]	1.59 [–2.04 to 5.22]	3.31 [0.55 to 6.08]^a^	3.22 [0.18 to 6.25]^a^	1.63 [–1.93 to 5.19]	1.74 [–2.17 to 5.64]
R2 (arb. unit)	3.29 [–0.56 to 7.13]	4.01 [–0.33 to 8.34]	1.83 [–2.06 to 5.73]	1.36 [–2.87 to 5.59]	2.78 [0.11 to 5.46]^a^	2.56 [–0.33 to 5.46]	1.00 [–3.44 to 5.43]	1.25 [–3.68 to 6.18]
R3 (arb. unit)	2.93 [0.03 to 5.83]^a^	3.51 [0.31 to 6.72]^a^	1.42 [–1.15 to 3.99]	0.89 [–1.76 to 3.54]	2.71 [0.72 to 4.69]^a^	2.51 [0.35 to 4.68]^a^	1.20 [–1.43 to 3.83]	1.44 [–1.49 to 4.36]
R4 (arb. unit)	1.92 [–0.86 to 4.70]	2.18 [–0.91 to 5.27]	0.37 [–1.91 to 2.65]	0.24 [–2.25 to 2.73]	1.42 [–0.35 to 3.19]	1.26 [–0.68 to 3.20]	0.97 [–1.22 to 3.15]	0.66 [–1.79 to 3.11]
R5 (arb. unit)	0.49 [–0.24 to 1.22]	0.60 [–0.18 to 1.38]	0.41 [–0.33 to 1.15]	0.33 [–0.45 to 1.12]	0.54 [–0.03 to 1.11]	0.49 [–0.12 to 1.10]	0.18 [–0.51 to 0.87]	0.14 [–0.60 to 0.88]

Abbreviations: arb. unit, arbitrary unit; TEWL, transepidermal water loss.

^a^ Statistically significant differences between groups (*p* < 0.05) when analyzed using ANCOVA, adjusted for baseline values and compliance rate.

**FIGURE 3:**
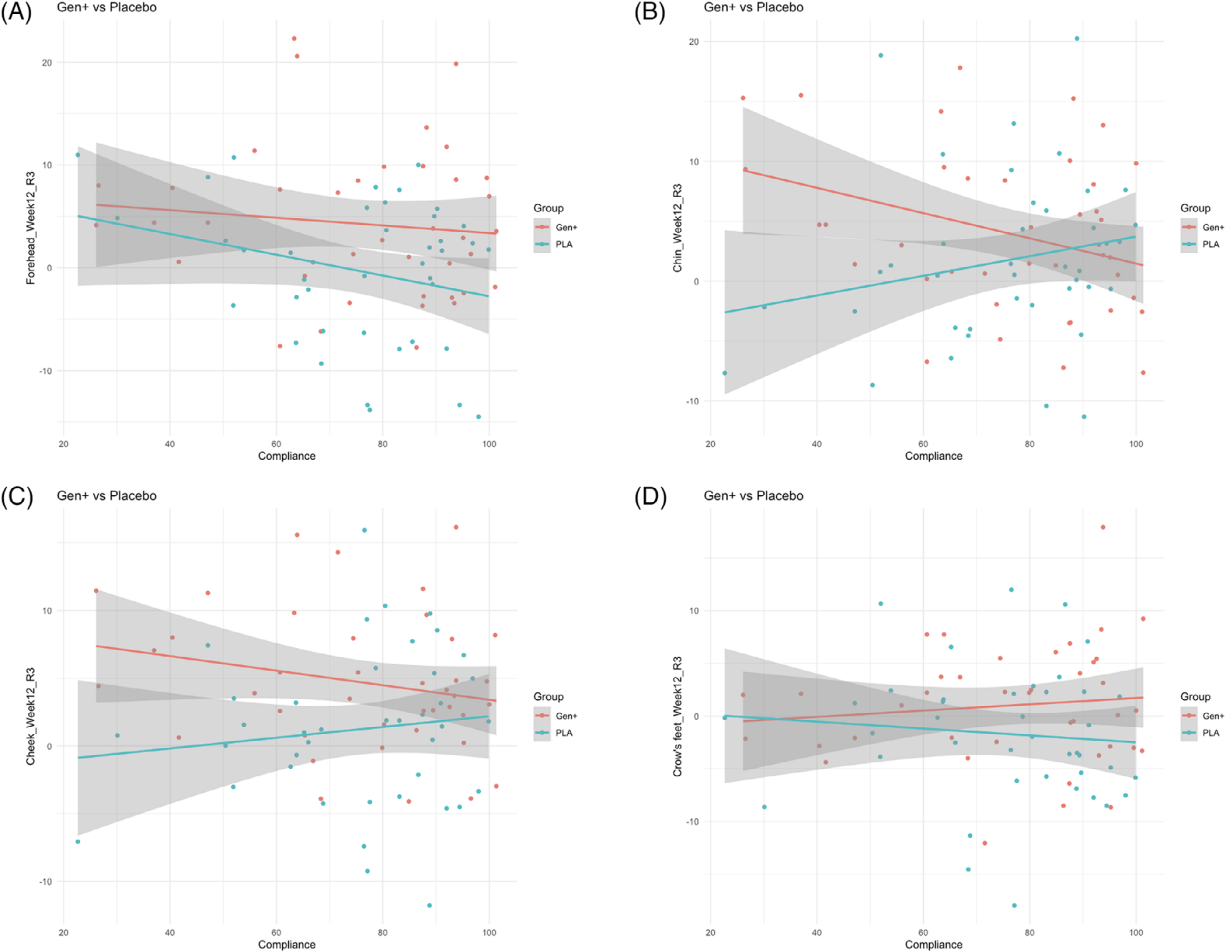
Regression analysis of the mean change from baseline in skin roughness (R3) following a 12‐week intervention with respect to compliance rate: (A) Forehead, (B) Chin, (C) Cheek, and (D) Crow's feet.

Furthermore, regression analysis was performed on the R3 parameter, which represents the average skin roughness and is considered a suitable indicator of overall roughness. The results indicated a decreasing trend in the mean change at Week 12 with higher compliance rates (%). This trend was observed across the forehead, chin, and cheeks, with a statistically significant reduction specifically in the chin area (Figure 3).

The time‐interaction analysis of facial skin properties parameters, including skin elasticity, hydration, transepidermal water loss, and wrinkle parameters, showed no significant differences across various locations when comparing Week 4 or 8 to the baseline. The mean differences between the GEN^+^ and PLA groups at Weeks 4 and 8 for facial skin property parameters are reported in Table S3.

### 3.3 Adverse Events

During the 12‐week study period, four participants in the GEN^+^ group and two participants in the PLA group experienced mild, self‐resolved skin rashes or itching. Additionally, other symptoms, such as burning sensations and peeling facial skin, were reported in seven participants in the GEN^+^ group and nine participants in the PLA group. For those experiencing rashes or peeling skin, the product (either GEN^+^ or PLA) was withheld until the symptoms were completely resolved. However, all participants who reported these issues resumed using the product and continued until the end of the study. No further skin rashes occurred after re‐exposure to the GEN^+^ product.

### 3.4 Participant Satisfaction

In terms of participant satisfaction with the product and skin outcomes after the 12‐week intervention, no significant differences were observed between the two groups. However, the GEN^+^ group demonstrated a higher satisfaction trend in both product satisfaction and satisfaction with their own skin outcomes across most evaluated domains. Detailed scores for each domain in both groups are provided in Table S4.

## 4 Discussion

In this study, improvements were observed in several aspects of facial skin appearance in men following the application of the GEN^+^ product. To understand the biological basis of these effects, it is important to consider the role of estrogen receptors (ERs), which are involved in various complex physiological processes. ERs exist in two subtypes, ERα and ERβ, each with distinct tissue distributions [36]. Both subtypes are expressed in human skin, but ERβ is the predominant isoform and is believed to mediate most of the estrogen‐related effects in cutaneous tissue, with no significant differences in ER expression levels between males and females [37].

Genistein, a naturally occurring phytoestrogen, exerts its biological effects primarily through estrogen receptors (ERs), exhibiting a selective affinity for ERβ [23, 38]. Following topical application, genistein can permeate the stratum corneum, allowing it to interact with cutaneous ERs [23, 39]. Given this mechanism, genistein is expected to exert comparable local effects on the skin across sexes. Furthermore, previous studies indicate that systemic absorption of genistein via topical administration is minimal, with the compound predominantly retained within the skin [40]. Consequently, the likelihood of systemic hormonal effects resulting from cutaneous exposure to genistein is considered low.

The concentration of genistein used in this study was 4%, a dosage that has been extensively investigated in prior clinical research. Evidence demonstrates that this concentration is effective in enhancing multiple aspects of skin health, including improvements in elasticity and moisture content, increased dermal vascularization, and greater epidermal thickness. Furthermore, significant increases in dermal levels of type I and III collagen, hyaluronic acid, glycosaminoglycans, and fibroblast density have been reported. Additionally, no significant local or systemic adverse events were reported. Collectively, these studies confirm both the clinical efficacy and favorable safety profile of genistein [35, 41, 42, 43, 44].

The formulation's overall efficacy is further supported by the inclusion of multiple bioactive ingredients such as genistein, bakuchiol, key vitamins (Vitamin C, Vitamin E, and Vitamin B3), and ceramides. All ingredients used in the formulation comply with applicable requirements and are not classified as prohibited or restricted under current cosmetic regulations in Thailand or according to ASEAN Cosmetic Harmonization standards [45, 46]. Collectively, these findings highlight the potential of such formulations to effectively address male skincare needs.

A key strength of this study lies in its robust methodology, which employed a high‐quality randomized controlled trial (RCT) design. The clinical trial adhered to the gold standard for reliability by using a randomized, double‐blind, placebo‐controlled approach. Study products were coded and randomly assigned, ensuring that both participants and investigators remained blind throughout the trial. Facial skin outcomes—including facial color, skin tone, spots, melanin and erythema levels, acne parameters, elasticity, hydration, skin barrier integrity, and wrinkles—were assessed objectively and quantitatively using reliable tools. RCT design is particularly advantageous in dermatological studies due to the high variability in skin measurements, which are influenced by environmental factors. For example, a previous study found a statistical correlation between air temperature and other environmental variables, such as relative humidity and air pressure. Specifically, skin elasticity was positively correlated with air temperature but negatively correlated with air pressure [47]. By including a placebo‐controlled group and randomization, RCTs mitigate the impact of these external variables, enhancing the validity of the findings. In contrast, single‐arm trials lack a control group, making it difficult to account for environmental influences and other confounding factors. This limitation complicates the interpretation of results and reduces the quality of evidence. Therefore, the rigorous RCT design employed in this study strengthens the validity and reliability of the observed outcomes.

Our analysis revealed that men could benefit from the GEN^+^ product in terms of skin appearance. Significant improvements were observed in skin lightening, color consistency, and spot count reduction, while the melanin index showed a decreasing trend across most facial areas following the 12‐week intervention with the GEN^+^ product. These improvements may be attributed to the various active ingredients in the GEN^+^ product, which target four primary stages of melanogenesis.

First, melanogenesis, the process of melanin production, begins with the activation of melanocytes, which is primarily influenced by ultraviolet (UV) radiation. UV exposure generates reactive oxygen species (ROS), leading to oxidative stress and inflammation that accelerate skin dyschromia and aging [13]. However, antioxidants can neutralize ROS, thereby providing photoprotective benefits [48]. The GEN^+^ formulation contains several antioxidants, including genistein, a predominant isoflavone in soybeans, and bakuchiol, both of which mitigate oxidative stress [49, 50, 51]. Additionally, Vitamin C and Vitamin E are well‐known antioxidants with anti‐inflammatory effects through several mechanisms, including the neutralization of ROS [52, 53], inhibiting NF‐κB signaling [53], and synergistic replenishment of the skin's antioxidant capacity [54]. Second, following melanocyte activation, tyrosine, the precursor substance, is converted into melanin through tyrosinase activity. Several components in the GEN^+^ formulation play a crucial role in this step, including genistein, which has demonstrated evidence of inhibiting tyrosinase [55]. Bakuchiol further regulates this process by interfering with α‐melanocyte‐stimulating hormone (α‐MSH) signaling [56]. The mechanism α‐MSH activates the melanocortin‐1 receptor (MC1R) and downstream cAMP signaling, which are essential for tyrosinase activity and melanogenesis [57]. Additionally, bakuchiol has been shown to competitively inhibit tyrosinase activity and may downregulate tyrosinase transcription and related proteins [58, 59]. Vitamin C also plays a crucial role in this step by interacting with copper ions at the tyrosinase‐active site, thereby inhibiting tyrosinase activity and reducing melanin formation [60]. Third, once melanin is produced, melanosomes containing melanin are transferred from melanocytes to keratinocytes, contributing to visible pigmentation. However, both bakuchiol and niacinamide (Vitamin B3) have been shown to inhibit melanosome transfer, effectively reducing pigment deposition in the epidermis [58, 61, 62]. Finally, pigment distribution and degradation are regulated through keratinocyte turnover and epidermal renewal. Vitamin C supports epidermal turnover, improving skin brightness and promoting a more consistent complexion [63]. Furthermore, the overall improvement in skin appearance observed with the GEN^+^ formulation may be attributed to the synergistic effects of its combined active ingredients. Previous clinical trials on nutraceutical products combining genistein with vitamins E and B3 have demonstrated improvements in facial skin quality [35]. Another study evaluating a serum containing bakuchiol, ascorbyl tetraisopalmitate (a stable vitamin C derivative), and melatonin reported significant clinical anti‐aging effects [64]. Additionally, strong evidence supports the synergistic benefits of combining topical vitamins C and E, which have been shown to provide superior photoprotection compared to the use of either vitamin alone [65].

Skin lightening, color uniformity, and spot count were assessed using Visioface, which enables consistent tracking of predefined facial marker areas over the study intervention. In contrast, parameters such as melanin and erythema were measured using Mexameter, a point‐based device. To minimize measurement bias, regions exhibiting pronounced pigmentation or inflammation (e.g., areas affected by active melasma or erythema) were intentionally excluded from Mexameter assessments. As a result, the baseline values recorded may have been lower, potentially limiting the detectable changes. While the melanin index showed a decreasing trend across most facial areas, the change did not reach statistical significance.

The GEN^+^ product demonstrated a trend toward promoting healthy skin, as indicated by increased skin hydration and a corresponding decrease in transepidermal water loss, suggesting enhanced skin barrier function after the 12‐week intervention. In terms of skin hydration, genistein has been shown to increase hyaluronic acid concentrations in the skin [44]. Hyaluronic acid appears to be a critical component of the extracellular matrix and functions as a biological humectant, which can retain moisture within the skin [66]. These findings align with previous studies, all of which observed improvements in skin hydration following the topical application of soy‐based products [67, 68, 69]. For TEWL, evidence suggests that improvements in TEWL and, consequently, skin barrier integrity are more pronounced with topical soy application than with oral supplementation [70]. Another key ingredient in GEN^+^ is ceramides. Incorporating ceramides into skincare formulations has been shown to directly contribute to the skin's lipid matrix, as ceramides are essential for forming multilamellar structures in the stratum corneum [34]. The presence of covalently bound ceramides is crucial for maintaining skin barrier integrity and moisture retention [71], supporting the observed improvements in both parameters.

In our study, no significant difference in skin elasticity was observed between the GEN^+^ and PLA groups. Skin elasticity naturally declines with age due to the deterioration of elastic fibers and changes in the dermis [72]. The lack of improvement in elasticity may be attributed to intrinsic aging and external factors, such as UV exposure and lifestyle choices [73], which can diminish the effects of natural treatments. This finding aligns with previous studies that found no association between dietary soy intake and skin elasticity [74]. Skin elasticity is a critical factor in wrinkle formation as it reflects the skin's ability to return to its original shape after deformation. Declining elasticity is associated with reduced skin resilience and increased wrinkle formation, particularly after age 40 [75], which aligns with the target age group of our study. Additionally, both groups experienced an increase in skin roughness during the 12‐week study period. Wrinkle parameters are influenced by multiple factors, with genetic predispositions in males often leading to deeper wrinkles than in females [11]. Furthermore, external factors such as environmental stressors and UV exposure are important contributors, as they can accelerate the breakdown of skin components and promote apoptosis in both epithelial and dermal cells [76]. Men are generally less likely to adopt sun‐protective behaviors, such as regular sunscreen use, which may further exacerbate wrinkle formation [18]. However, regression analysis of R3 (average roughness parameters) indicated a decreasing trend in roughness with higher compliance levels. This suggests that participant compliance with the product strongly influenced wrinkle parameters. Nonetheless, significant variability in compliance rates among participants limits the ability to draw definitive conclusions.

Moreover, the physicochemical properties of the ingredients in the GEN^+^ product play a crucial role in their efficacy on the skin. Genistein and bakuchiol exhibit high partition coefficients [77, 78], indicating their hydrophobic nature and tendency to concentrate in lipid phases [79]. Consequently, in vitro evidence on skin permeation and retention has revealed that while genistein penetrates the outermost skin layer, the stratum corneum (SC), only a small amount is detected in the deeper layers, such as the viable epidermis and dermis [80], potentially limiting its dermal bioavailability. This may explain why parameters assessing deeper skin properties do not show significant improvement compared to the control group. However, since most melanocytes reside in the viable epidermis, where melanin production occurs [81], genistein and bakuchiol can effectively target these cells and modulate melanin synthesis and distribution. This results in a significant reduction in pigmentation, making these compounds effective for skin lightening and producing visible changes in skin appearance, as reflected in parameters such as skin tone and pigmentation uniformity.

The GEN^+^ product was well tolerated throughout the 12‐week application period, with 11 participants in each group reporting mild rashes or slight skin peeling. These adverse events were self‐resolving in all cases. The reactions were attributed to factors such as extreme outdoor activities, facial scrubbing, or over‐exfoliation rather than the product itself. Notably, the rashes did not recur upon reapplication, further supporting the product's safety profile.

Although this study has several strengths, as mentioned above, certain limitations should be acknowledged. First, variability in participant compliance and daily activities may have influenced the outcomes. Our study did not fully control for diet, which could have introduced variations, as certain foods, such as carotenoids and polyphenols, are known to provide photoprotective benefits and may have affected the skin [82, 83, 84]. Nonetheless, the results may still reflect an individual's typical diet outside a clinical research setting. Second, the study primarily focused on facial skin, leaving the potential effects of the GEN^+^ product on other body areas unexplored. Third, this study did not investigate the benefits of the GEN^+^ product at the molecular level. Future research should address these limitations by improving compliance monitoring, evaluating the product's effects on additional skin areas, and applying molecular analyses to explore its mechanisms at the cellular level. Incorporating advanced technologies for assessing facial skin lesions and conditions would also provide deeper insights into their effects.

## 5 Conclusion

In conclusion, the findings highlight the potential benefits of the Genistein Plus Bakuchiol and Vitamins (GEN^+^) product for male facial skin. The product demonstrated improvements in skin appearance, including enhanced skin lightening, improved color consistency, and a reduction in spot count among male participants. These outcomes are especially relevant for addressing key concerns in skin aging, which are prevalent issues in aging societies worldwide, particularly in regions with intense year‐round UV radiation. Additionally, the GEN^+^ product was well‐tolerated and safe. These results provide a valuable foundation for the further development of skincare products designed to meet the specific needs of men.

## Funding

This research was supported by the CMU Mid‐Career Research Fellowship program (MRCMU2566R_009 and MRCMU2567‐2_008).

## Ethics Statement

Ethical approval was obtained from the Research Ethics Committee of the Faculty of Medicine, Chiang Mai University (No. 020/2024).

## Conflicts of Interest

K.Y., J.A., P.K., P.A., R.I., T.K., J.C., and K.E. declare no competing interests. Chiang Mai University, a public university, has submitted a petty patent application for the GEN^+^ product, with M.N., N.K., and S.T. listed as the inventors.

## Supporting information




**Supplementary Table 1**. Participants’ baseline characteristics.
**Supplementary Table 2**. Mean differences between the GEN^+^ and PLA groups at Weeks 4 and 8 for facial skin appearance parameters, including skin color, spot count, melanin, erythema, and porphyrins. The analysis comprised an intention‐to‐treat (ITT) analysis (n = 80; GEN^+^ = 40, PLA = 40) and a per‐protocol (PP) analysis of participants with compliance greater than 50% of the prescribed dosage (n = 71; GEN^+^ = 34, PLA = 37). The results were reported as mean differences (MD) with 95% confidence intervals (CI).
**Supplementary Table 3**. Mean differences between the GEN^+^ and PLA groups at Weeks 4 and 8 for facial skin properties parameters, including facial skin elasticity, hydration, transepidermal water loss, and wrinkles. The analysis comprised an intention‐to‐treat (ITT) analysis (n = 80; GEN^+^ = 40, PLA = 40) and a per‐protocol (PP) analysis of participants with compliance greater than 50% of the prescribed dosage (n = 71; GEN^+^ = 34, PLA = 37). The results were reported as mean differences (MD) with 95% confidence intervals (CI).
**Supplementary Table 4**. Participants’ satisfaction with the product and skin outcomes after the 12‐week intervention.

## Data Availability

The data that support the findings of this study are available from the corresponding author upon reasonable request.
