# Supplementary material for: A Randomized Controlled Trial of the Genistein Plus Bakuchiol and Vitamins (GEN+) Product for Male Facial Skin: Effects on Skin Appearance and Properties
Source: Skin Res Technol. 2026 Jan 21;32(1):e70310. doi: 10.1111/srt.70310 (PMC12821225; doi:10.1111/srt.70310)
Supplement: Supplementary file 1 — Supplementary Table 1. Participants’ baseline characteristics. Supplementary Table 2. Mean differences between the GEN+ and PLA groups at Weeks 4 and 8 for facial skin appearance parameters, including skin color, spot count, melanin, erythema, and porphyrins. The analysis comprised an intention‐to‐treat (ITT) analysis (n = 80; GEN+ = 40, PLA = 40) and a per‐protocol (PP) analysis of participants with compliance greater than 50% of the prescribed dosage (n = 71; GEN+ = 34, PLA = 37). The results were reported as mean differences (MD) with 95% confidence intervals (CI). Supplementary Table 3. Mean differences between the GEN+ and PLA groups at Weeks 4 and 8 for facial skin properties parameters, including facial skin elasticity, hydration, transepidermal water loss, and wrinkles. The analysis comprised an intention‐to‐treat (ITT) analysis (n = 80; GEN+ = 40, PLA = 40) and a per‐protocol (PP) analysis of participants with compliance greater than 50% of the prescribed dosage (n = 71; GEN+ = 34, PLA = 37). The results were reported as mean differences (MD) with 95% confidence intervals (CI). Supplementary Table 4. Participants’ satisfaction with the product and skin outcomes after the 12‐week intervention. [file SRT-32-e70310-s001.docx]

**Supplementary Table I** Participants’ baseline characteristics

| **Locations**  **Parameters** | **Forehead** | | **Chin** | | **Cheek**  **(average Left and Right side)** | | **Nose** | |
| --- | --- | --- | --- | --- | --- | --- | --- | --- |
|  | **GEN^+^ Group**  **(n = 40)** | **PLA Group**  **(n = 40)** | **GEN^+^ Group**  **(n = 40)** | **PLA Group**  **(n = 40)** | **GEN^+^ Group**  **(n = 40)** | **PLA Group**  **(n = 40)** | **GEN^+^ Group**  **(n = 40)** | **PLA Group**  **(n = 40)** |
|  | Mean ± SD | Mean ± SD | Mean ± SD | Mean ± SD | Mean ± SD | Mean ± SD | Mean ± SD | Mean ± SD |
| **Facial Skin Color and Spot Parameters** | | | | | | | | |
| ΔL1 | -1.18 ± 2.44 | -1.73 ± 2.51 | -0.68 ± 2.12 | -1.35 ± 2.51 | -2.16 ± 2.35 | -1.69 ± 2.01 |  |  |
| ΔL2 | -0.43 ± 2.46 | -1.13 ± 2.07 | 0.23 ± 1.46 | -0.85 ± 2.20 | -1.11 ± 3.09 | -1.79 ± 2.64 |  |  |
| ΔE1 | 3.20 ± 1.92 | 3.15 ± 2.15 | 2.40 ± 1.57 | 2.85 ± 2.30 | 4.31 ± 1.79 | 3.68 ± 1.84 |  |  |
| ΔE2 | 2.53 ± 1.74 | 2.35 ± 1.85 | 1.78 ± 1.10 | 2.35 ± 1.72 | 4.25 ± 1.94 | 4.01 ± 1.67 |  |  |
| Spot (count) | 1.60 ± 1.43 | 1.93 ± 3.76 | 1.38 ± 1.33 | 1.70 ± 2.03 | 3.20 ± 2.03 | 4.20 ± 2.95 |  |  |
| Spot area (px) | 1291.33 ± 1374.29 | 1588.80 ± 3634.58 | 1033.63 ± 1049.28 | 1357.05 ± 1739.21 | 2858.23 ± 1949.58 | 3839.86 ± 2942.91 |  |  |
| **Skin color parameters** | | | | | | | | |
| Avg. L (arb. unit) | 51.97 ± 3.09 | 51.97 ± 2.62 | 51.00 ± 2.66 | 50.07 ± 2.63 | 52.78 ± 3.05 | 52.70 ± 2.78 | 52.49 ± 2.74 | 51.83 ± 2.74 |
| Avg. A (arb. unit) | 11.95 ± 1.27 | 11.98 ± 1.38 | 12.56 ± 1.78 | 12.65 ± 1.79 | 10.91 ± 1.38 | 11.10 ± 1.30 | 13.35 ± 1.76 | 13.25 ± 1.51 |
| Avg. B (arb. unit) | 14.75 ± 1.41 | 14.60 ± 1.56 | 15.14 ± 1.48 | 14.74 ± 1.62 | 15.18 ± 1.24 | 14.57 ± 1.51 | 14.80 ± 1.40 | 14.74 ± 1.49 |
| Avg. ITA (arb. unit) | 7.48 ± 12.10 | 7.59 ± 10.11 | 3.83 ± 9.94 | 0.01 ± 10.17 | 10.06 ± 11.25 | 10.52 ± 10.7 | 9.35 ± 10.37 | 7.15 ± 10.66 |
| **Melanin and Erythema parameters** | | | | | | | | |
| Melanin | 320.58 ± 78.36 | 326.13 ± 67.40 | 317.25 ± 62.69 | 339.18 ± 70.45 | 298.58 ± 65.71 | 298.28 ± 65.84 | 304.47 ± 58.56 | 318.81 ± 62.40 |
| Erythema | 432.24 ± 57.28 | 431.70 ± 49.61 | 447.18 ± 56.32 | 450.99 ± 64.35 | 392.39 ± 57.34 | 399.96 ± 47.49 | 488.46 ± 52.17 | 487.98 ± 46.72 |
| **Porphyrins parameters** | | | | | | | | |
| Size (%) | 0.74 ± 0.97 | 0.87 ± 1.11 | 1.48 ± 1.10 | 1.69 ± 1.22 | 0.77 ± 0.69 | 0.86 ± 0.67 |  |  |
| Count | 13.39 ±  15.13 | 14.98 ± 16.76 | 24.27 ± 13.54 | 27.21 ± 14.90 | 14.01 ± 10.44 | 17.44 ± 11.97 |  |  |
| Avg. Intensity | 155.15 ± 42.22 | 154.84 ± 49.54 | 168.85 ± 24.08 | 162.84 ± 34.52 | 173.23 ± 32.80 | 173.09 ± 29.60 |  |  |
| **Skin elasticity parameters** | | | | | | | | |
| R0 | 0.31 ± 0.07 | 0.36 ± 0.09 | 0.33 ± 0.09 | 0.37 ± 0.08 | 0.38 ± 0.05 | 0.39 ± 0.06 |  |  |
| R2 | 59.72 ± 7.48 | 59.20 ± 12.64 | 57.88 ± 8.02 | 61.33 ± 9.38 | 58.98 ± 5.79 | 59.61 ± 7.35 |  |  |
| R5 | 45.23 ± 6.40 | 42.68 ± 10.15 | 49.89 ± 7.37 | 51.95 ± 9.04 | 40.54 ± 4.54 | 41.15 ± 6.12 |  |  |
| R7 | 33.31 ± 4.92 | 31.41 ± 6.27 | 35.92 ± 6.49 | 37.53 ± 6.94 | 31.63 ± 3.91 | 31.95 ± 4.78 |  |  |
| **Skin hydration parameters** | | | | | | | | |
| Hydration value  (arb. unit) | 63.16 ± 8.69 | 61.34 ± 7.90 | 64.83 ± 8.54 | 62.24 ± 8.32 | 65.86 ± 7.62 | 63.29 ± 8.47 |  |  |
| **TEWL parameters** | | | | | | | | |
| TEWL | 19.51 ± 3.87 | 20.43 ± 6.02 | 26.18 ± 6.38 | 28.39 ± 6.57 | 15.36 ± 3.98 | 16.43 ± 4.60 |  |  |
| **Locations**  **Parameters** | **Forehead** | | **Chin** | | **Cheek**  **(average Left and Right side)** | | **Crow’s feet**  **(average Left and Right side)** | |
|  | **GEN^+^ Group**  **(n = 40)** | **PLA Group**  **(n = 40)** | **GEN^+^ Group**  **(n = 40)** | **PLA Group**  **(n = 40)** | **GEN^+^ Group**  **(n = 40)** | **PLA Group**  **(n = 40)** | **GEN^+^ Group**  **(n = 40)** | **PLA Group**  **(n = 40)** |
|  | Mean ± SD | Mean ± SD | Mean ± SD | Mean ± SD | Mean ± SD | Mean ± SD | Mean ± SD | Mean ± SD |
| **Skin wrinkle parameters** | | | | | | | | |
| R1  (arb. unit) | 71.08 ± 7.58 | 75.13 ± 8.76 | 81.97 ± 12.41 | 82.90 ± 8.76 | 78.86 ± 7.16 | 80.19 ± 8.39 | 76.26 ± 10.30 | 79.06 ± 7.94 |
| R2  (arb. unit) | 62.51 ± 7.31 | 65.81 ± 7.73 | 70.11 ± 10.11 | 71.69 ± 7.90 | 70.17 ± 6.36 | 71.93 ± 7.66 | 63.46 ± 7.07 | 66.02 ± 5.88 |
| R3  (arb. unit) | 48.11 ± 5.90 | 51.01 ± 6.05 | 54.67 ± 7.41 | 55.95 ± 6.37 | 55.42 ± 5.32 | 56.83 ± 6.19 | 47.55 ± 5.81 | 49.75 ± 4.53 |
| R4  (arb. unit) | 38.92 ± 4.66 | 41.29 ± 4.98 | 43.27 ± 6.55 | 44.00 ± 4.88 | 40.85 ± 4.00 | 41.79 ± 4.31 | 44.82 ± 5.45 | 46.60 ± 4.46 |
| R5  (arb. unit) | 9.12 ± 1.11 | 9.72 ± 1.41 | 12.00 ± 2.27 | 11.87 ± 1.70 | 10.89 ± 1.44 | 10.95 ± 1.50 | 12.25 ± 2.36 | 12.59 ± 1.84 |

**Abbreviations:** px, pixels; arb. unit, arbitrary unit; Avg., average; TEWL, transepidermal water loss

**Supplementary Table II** Mean differences between the GEN^+^ and PLA groups at Weeks 4 and 8 for facial skin appearance parameters, including skin color, spot count, melanin, erythema, and porphyrins. The analysis comprised an intention-to-treat (ITT) analysis (n = 80; GEN^+^ = 40, PLA = 40) and a per-protocol (PP) analysis of participants with compliance greater than 50% of the prescribed dosage (n = 71; GEN^+^ = 34, PLA = 37). The results were reported as mean differences (MD) with 95% confidence intervals (CI).

| **Locations**  **Parameters** | **Forehead** | | **Chin** | | **Cheek**  **(average Left and Right side)** | | **Nose** | | |
| --- | --- | --- | --- | --- | --- | --- | --- | --- | --- |
|  | **ITT** | **PP** | **ITT** | **PP** | **ITT** | **PP** | **ITT** | **PP** | |
| **Week 4** | | | | | | | | | |
| **Facial Skin Color and Spot Parameters** | | | | | | | | | |
| ΔL1 | 0.10  [-0.28 to 0.47] | 0.13  [-0.26 to 0.53] | 0.05  [-0.50 to 0.60] | -0.16  [-0.91 to 0.60] | 0.24  [-0.24 to 0.72] | 0.23  [-0.28 to 0.74] |  | |  |
| ΔL2 | 0.15  [-0.32 to 0.62] | 0.19  [-0.29 to 0.66] | 0.76  [0.12 to 1.41]^*^ | -0.35  [-1.14 to 0.45] | 0.62  [0.09 to 1.16]^*^ | 0.69  [0.13 to 1.26]^*^ |  | |  |
| ΔE1 | -0.22  [-0.66 to 0.22] | -0.36  [-0.84 to 0.12] | -0.46  [-1.10 to 0.17] | 0.16  [-0.47 to 0.79] | -0.57  [-1.12 to -0.02]^*^ | -0.60  [-1.20 to 0.00]^*^ |  | |  |
| ΔE2 | 0.19  [-0.28 to 0.65] | 0.07  [-0.41 to 0.55] | 0.11  [-0.55 to 0.78] | 0.41  [-0.10 to 0.92] | -0.05  [-0.52 to 0.42] | -0.16  [-0.66 to 0.35] |  | |  |
| Spot (count) | -0.68  [-1.37 to 0.02] | -1.03  [-1.79 to -0.27]^*^ | 0.72  [-0.10 to 1.54] | -0.57  [-1.21 to 0.07] | 0.35  [-0.31 to 1.02] | 0.26  [-0.48 to 1.00] |  | |  |
| Spot area (px) | -99.82  [-690.38 to 490.74] | -344.99  [-994.43 to 304.46] | 474.55  [-335.85 to 1284.94] | -637.85  [-1157.47 to  -118.22]^*^ | 403.57  [-217.62 to 1024.76] | 313.44  [-351.48 to 978.35] |  | |  |
| **Skin color parameters** | | | | | | | | | |
| Avg L (arb. unit) | -0.17  [-0.71 to 0.37] | -0.18  [-0.77 to 0.42] | -0.25  [-0.76 to 0.27] | -0.24  [-0.82 to 0.34] | 0.19  [-0.27 to 0.64] | 0.26  [-0.21 to 0.74] | 0.09  [-0.62 to 0.80] | | -0.04  [-0.81 to 0.73] |
| Avg A (arb. unit) | 0.05  [-0.35 to 0.46] | 0.10  [-0.32 to 0.53] | -0.01  [-0.37 to 0.36] | 0.08  [-0.32 to 0.47] | 0.03  [-0.36 to 0.41] | 0.00  [-0.43 to 0.43] | -0.06  [-0.63 to 0.51] | | -0.18  [-0.83 to 0.46] |
| Avg B (arb. unit) | 0.03  [-0.41 to 0.47] | 0.00  [-0.49 to 0.48] | -0.28  [-0.68 to 0.13] | -0.39  [-0.83 to 0.05] | -0.02  [-0.39 to 0.35] | -0.07  [-0.48 to 0.35] | -0.21  [-0.67 to 0.25] | | -0.15  [-0.68 to 0.38] |
| Avg. ITA  (arb. unit) | -0.66  [-2.77 to 1.46] | -0.70  [-3.04 to 1.63] | -1.33  [-3.30 to 0.64] | -1.27  [-3.49 to 0.96] | 0.82  [-1.00 to 2.64] | 1.24  [-0.71 to 3.19] | 0.55  [-1.96 to 3.06] | | 0.03  [-2.69 to 2.74] |
| **Melanin and Erythema parameters** | | | | | | | | | |
| Melanin | 0.22  [-10.30 to 10.73] | 1.12  [-10.37 to 12.62] | 0.16  [-9.63 to 9.95] | -0.10  [-10.78 to 10.58] | -1.96  [-12.21 to 8.29] | -4.30  [-15.63 to 7.03] | -4.34  [-16.25 to 7.57] | | -4.98  [-18.13 to 8.17] |
| Erythema | 0.32  [-10.52 to 11.16] | 2.40  [-9.66 to 14.46] | -2.28  [-15.39 to 10.82] | 0.48  [-13.72 to 14.68] | 3.49  [-7.67 to 14.65] | 5.15  [-7.43 to 17.72] | 2.02  [-8.18 to 12.23] | | -1.30  [-12.21 to 9.62] |
| **Porphyrins parameters** | | | | | | | | | |
| Size (%) | -0.20  [-0.48 to 0.07] | -0.23  [-0.54 to 0.08] | -0.01  [-0.30 to 0.28] | 0.02  [-0.30 to 0.34] | -0.02  [-0.20 to 0.17] | -0.01  [-0.21 to 0.20] |  | |  |
| Count | -4.76  [-8.03 to -1.48]^*^ | -4.63  [-8.27 to -0.99]^*^ | -1.71  [-5.66 to 2.23] | -1.71  [-6.12 to 2.71] | -0.28  [-2.81 to 2.25] | -0.68  [-3.52 to 2.15] |  | |  |
| Avg. Intensity | -6.89  [-27.11 to 13.34] | -1.42  [-21.64 to 18.80] | -0.53  [-6.76 to 5.71] | -0.06  [-5.79 to 5.68] | 0.08  [-6.35 to 6.51] | -1.07  [-8.24 to 6.09] |  | |  |
| **Week 8** | | | | | | | | | |
| **Facial Skin Color and Spot Parameters** | | | | | | | | | |
| ΔL1 | 0.31  [-0.16 to 0.77] | 0.21  [-0.27 to 0.69] | 0.21  [-0.33 to 0.75] | 0.21  [-0.53 to 0.94] | 0.29  [-0.20 to 0.79] | 0.35  [-0.19 to 0.89] |  | |  |
| ΔL2 | 0.07  [-0.54 to 0.67] | 0.21  [-0.41 to 0.84] | 0.65  [0.04 to 1.25]^*^ | -0.15  [-0.97 to 0.67] | 0.50  [-0.05 to 1.06] | 0.72  [0.11 to 1.33]^*^ |  | |  |
| ΔE1 | -0.13  [-0.64 to 0.37] | -0.12  [-0.68 to 0.44] | -0.31  [-0.84 to 0.22] | 0.19  [-0.45 to 0.83] | -0.49  [-0.99 to 0.00] | -0.59  [-1.12 to -0.06]^*^ |  | |  |
| ΔE2 | 0.03  [-0.50 to 0.57] | 0.13  [-0.44 to 0.69] | 0.09  [-0.51 to 0.68] | 0.04  [-0.59 to 0.67] | -0.11  [-0.60 to 0.39] | -0.14  [-0.69 to 0.40] |  | |  |
| Spot (count) | -0.55  [-1.13 to 0.03] | -0.77  [-1.36 to -0.18]^*^ | 0.58  [-0.30 to 1.47] | -0.01  [-0.68 to 0.66] | 0.28  [-0.39 to 0.94] | 0.41  [-0.34 to 1.16] |  | |  |
| Spot area (px) | -348.43  [-916.51 to 219.66] | -480.92  [-1101.41 to 139.56] | 257.57  [-764.65 to 1279.79] | 66.06  [-557.67 to 689.79] | 247.55  [-435.36 to 930.47] | 315.59  [-387.09 to 1018.28] |  | |  |
| **Skin color parameters** | | | | | | | | | |
| Avg L (arb. unit) | -0.41  [-0.91 to 0.09] | -0.38  [-0.93 to 0.17] | 0.42  [-0.13 to 0.97] | 0.64  [0.04 to 1.23]^*^ | 0.42  [0.00 to 0.83] | 0.45  [-0.02 to 0.91] | -0.05  [-0.72 to 0.63] | | -0.15  [-0.91 to 0.60] |
| Avg A (arb. unit) | 0.12  [-0.30 to 0.55] | 0.08  [-0.36 to 0.52] | -0.23  [-0.62 to 0.16] | -0.30  [-0.68 to 0.08] | 0.01  [-0.34 to 0.35] | -0.07  [-0.44 to 0.30] | -0.03  [-0.54 to 0.49] | | -0.08  [-0.61 to 0.46] |
| Avg B (arb. unit) | 0.09  [-0.29 to 0.48] | 0.13  [-0.30 to 0.55] | -0.09  [-0.50 to 0.32] | 0.05  [-0.38 to 0.49] | 0.03  [-0.34 to 0.40] | 0.06  [-0.37 to 0.48] | 0.09  [-0.36 to 0.53] | | 0.24  [-0.25 to 0.72] |
| Avg. ITA  (arb. unit) | -1.54  [-3.46 to 0.38] | -1.52  [-3.64 to 0.60] | 1.30  [-0.74 to 3.34] | 2.14  [-0.07 to 4.35] | 1.93  [0.20 to 3.66]^*^ | 2.21  [0.28 to 4.14]^*^ | 0.11  [-2.33 to 2.56] | | -0.34  [-3.07 to 2.39] |
| **Melanin and Erythema parameters** | | | | | | | | | |
| Melanin | 5.30  [-6.58 to 17.18] | 4.03  [-9.02 to 17.08] | 3.83  [-7.68 to 15.35] | 5.40  [-7.12 to 17.92] | -9.31  [-18.84 to 0.22] | -10.32  [-20.84 to 0.20] | -8.66  [-21.67 to 4.35] | | -6.47  [-19.92 to 6.98] |
| Erythema | 2.97  [-8.14 to 14.08] | 2.67  [-8.69 to 14.04] | -10.20  [-21.36 to 0.96] | -9.39  [-21.54 to 2.76] | 4.29  [-5.31 to 13.89] | 2.04  [-8.51 to 12.58] | -4.15  [-17.79 to 9.50] | | -5.18  [-20.08 to 9.72] |
| **Porphyrins parameters** | | | | | | | | | |
| Size (%) | -0.07  [-0.35 to 0.21] | -0.11  [-0.43 to 0.20] | 0.25  [-0.07 to 0.58] | 0.24  [-0.12 to 0.59] | 0.08  [-0.12 to 0.28] | 0.04  [-0.17 to 0.25] |  | |  |
| Count | -2.11  [-5.49 to 1.28] | -2.12  [-5.95 to 1.71] | 3.71  [-0.14 to 7.57] | 3.43  [-0.45 to 7.32] | 1.02  [-1.40 to 3.43] | 0.26  [-2.43 to 2.95] |  | |  |
| Avg. Intensity | -9.33  [-26.88 to 8.22] | -4.70  [-21.94 to 12.54] | -0.07  [-6.61 to 6.46] | -0.99  [-6.87 to 4.89] | -2.49  [-13.00 to 8.01] | -7.96  [-19.29 to 3.37] |  | |  |

* Statistically significant differences between groups (P < 0.05) when analyzed using ANCOVA, adjusted for baseline values and compliance rate.

**Abbreviations:** px, pixels; arb. unit, arbitrary unit; Avg., average

**Supplementary Table III** Mean differences between the GEN^+^ and PLA groups at Weeks 4 and 8 for facial skin properties parameters, including facial skin elasticity, hydration, transepidermal water loss, and wrinkles. The analysis comprised an intention-to-treat (ITT) analysis (n = 80; GEN^+^ = 40, PLA = 40) and a per-protocol (PP) analysis of participants with compliance greater than 50% of the prescribed dosage (n = 71; GEN^+^ = 34, PLA = 37). The results were reported as mean differences (MD) with 95% confidence intervals (CI).

| **Locations**  **Parameters** | **Forehead** | | **Chin** | | **Cheek**  **(average Left and Right side)** | | **Crow’s feet**  **(average Left and Right side)** | | |
| --- | --- | --- | --- | --- | --- | --- | --- | --- | --- |
|  | **ITT** | **PP** | **ITT** | **PP** | **ITT** | **PP** | **ITT** | **PP** | |
| **Week 4** | | | | | | | | | |
| **Skin elasticity parameters** | | | | | | | | | |
| R0 | 0.01  [-0.03 to 0.05] | 0.02  [-0.02 to 0.05] | 0.01  [-0.02 to 0.03] | 0.01  [-0.02 to 0.03] | 0.01  [-0.01 to 0.03] | 0.02  [0.00 to 0.04] |  | |  |
| R2 | -1.51  [-5.17 to 2.15] | -1.73  [-5.50 to 2.04] | -1.05  [-4.00 to 1.91] | -1.32  [-4.66 to 2.01] | -2.00  [-4.48 to 0.48] | -1.50  [-4.14 to 1.14] |  | |  |
| R5 | -2.40  [-5.81 to 1.00] | -2.81  [-6.16 to 0.54] | 0.82  [-2.10 to 3.74] | 1.58  [-1.56 to 4.71] | -2.27  [-4.63 to 0.09] | -2.13  [-4.73 to 0.47] |  | |  |
| R7 | -1.91  [-4.29 to 0.46] | -2.33  [-4.69 to 0.02] | -0.71  [-2.89 to 1.48] | -0.35  [-2.75 to 2.06] | -1.57  [-3.22 to 0.08] | -1.42  [-3.22 to 0.37] |  | |  |
| **Skin hydration parameters** | | | | | | | | | |
| Hydration value  (arb. unit) | 1.26  [-1.62 to 4.14] | 1.31  [-1.68 to 4.30] | -0.09  [-4.03 to 3.84] | 1.00  [-3.01 to 5.01] | 1.09  [-1.79 to 3.97] | 0.18  [-2.92 to 3.28] |  | |  |
| **TEWL parameters** | | | | | | | | | |
| TEWL | -1.42  [-3.68 to 0.84] | -2.04  [-4.42 to 0.34] | -0.88  [-4.68 to 2.92] | -1.24  [-5.45 to 2.97] | -1.26  [-3.30 to 0.78] | -1.42  [-3.69 to 0.85] |  | |  |
| **Skin wrinkle parameters** | | | | | | | | | |
| R1 (arb. unit) | 0.79  [-2.92 to 4.50] | 0.64  [-3.55 to 4.82] | 2.02  [-1.67 to 5.70] | 1.02  [-2.74 to 4.79] | 2.91  [0.03 to 5.79]^*^ | 2.29  [-0.77 to 5.34] | 2.02  [-0.69 to 4.73] | | 1.79  [-1.16 to 4.75] |
| R2 (arb. unit) | 0.49  [-2.89 to 3.87] | 0.34  [-3.49 to 4.16] | 1.62  [-1.46 to 4.70] | 0.79  [-2.35 to 3.93] | 2.25  [-0.34 to 4.85] | 1.64  [-1.03 to 4.32] | 1.79  [-0.66 to 4.24] | | 1.76  [-0.96 to 4.48] |
| R3 (arb. unit) | 0.29  [-2.49 to 3.07] | 0.10  [-3.05 to 3.25] | 1.02  [-1.40 to 3.44] | 0.35  [-2.18 to 2.88] | 2.10  [0.09 to 4.12]^*^ | 1.58  [-0.51 to 3.68] | 1.64  [-0.32 to 3.59] | | 1.76  [-0.41 to 3.94] |
| R4 (arb. unit) | -0.81  [-3.41 to 1.78] | -0.77  [-3.72 to 2.17] | 1.94  [-0.47 to 4.35] | 0.57  [-1.78 to 2.91] | 1.09  [-0.75 to 2.93] | 0.50  [-1.32 to 2.33] | 1.51  [-0.43 to 3.45] | | 1.20  [-0.95 to 3.36] |
| R5 (arb. unit) | -0.01  [-0.65 to 0.62] | -0.01  [-0.71 to 0.68] | 0.04  [-0.71 to 0.79] | 0.11  [-0.74 to 0.95] | 0.46  [-0.04 to 0.95] | 0.34  [-0.19 to 0.88] | 0.13  [-0.36 to 0.62] | | 0.03  [-0.47 to 0.54] |
| **Week 8** | | | | | | | | | |
| **Skin elasticity parameters** | | | | | | | | | |
| R0 | 0.00  [-0.04 to 0.04] | 0.01  [-0.04 to 0.05] | 0.02  [-0.01 to 0.06] | 0.02  [-0.01 to 0.05] | 0.01  [-0.01 to 0.04] | 0.02  [-0.01 to 0.04] |  | |  |
| R2 | 1.80  [-1.42 to 5.02] | 0.82  [-2.54 to 4.19] | -1.00  [-4.28 to 2.28] | -1.90  [-5.34 to 1.55] | -1.27  [-3.94 to 1.41] | -1.43  [-4.37 to 1.52] |  | |  |
| R5 | -0.07  [-3.51 to 3.37] | -0.78  [-4.41 to 2.85] | -2.24  [-5.19 to 0.71] | -2.12  [-5.26 to 1.01] | -0.72  [-3.15 to 1.71] | -1.01  [-3.46 to 1.45] |  | |  |
| R7 | -0.20  [-2.44 to 2.04] | -0.66  [-2.98 to 1.65] | -1.69  [-4.13 to 0.74] | -1.87  [-4.48 to 0.73] | -0.66  [-2.28 to 0.97] | -0.87  [-2.50 to 0.75] |  | |  |
| **Skin hydration parameters** | | | | | | | | | |
| Hydration value  (arb. unit) | -1.96  [-5.24 to 1.32] | -1.92  [-5.53 to 1.70] | 0.65  [-3.15 to 4.45] | 0.75  [-3.48 to 4.98] | -0.06  [-3.41 to 3.29] | -0.79  [-4.47 to 2.89] |  | |  |
| **TEWL parameters** | | | | | | | | | |
| TEWL | -1.08  [-3.15 to 1.00] | -1.45  [-3.71 to 0.80] | -1.93  [-5.52 to 1.67] | -2.73  [-6.71 to 1.24] | -0.44  [-2.61 to 1.73] | -0.91  [-3.28 to 1.47] |  | |  |
| **Skin wrinkle parameters** | | | | | | | | | |
| R1 (arb. unit) | 3.90  [-0.35 to 8.15] | 4.00  [-0.68 to 8.67] | 0.15  [-3.25 to 3.55] | -0.06  [-3.59 to 3.46] | 1.69  [-0.96 to 4.34] | 2.00  [-0.87 to 4.86] | 0.55  [-2.86 to 3.96] | | 0.25  [-3.33 to 3.83] |
| R2 (arb. unit) | 2.65  [-1.14 to 6.43] | 2.97  [-1.22 to 7.16] | 0.78  [-2.25 to 3.81] | 0.37  [-2.72 to 3.45] | 0.59  [-1.77 to 2.95] | 0.60  [-1.95 to 3.15] | 0.34  [-2.54 to 3.22] | | 0.35  [-2.58 to 3.28] |
| R3 (arb. unit) | 1.95  [-1.00 to 4.91] | 2.23  [-1.05 to 5.50] | 1.01  [-1.33 to 3.35] | 0.60  [-1.70 to 2.91] | 0.16  [-1.78 to 2.09] | 0.24  [-1.87 to 2.35] | 0.40  [-1.92 to 2.73] | | 0.64  [-1.75 to 3.03] |
| R4 (arb. unit) | 1.87  [-1.14 to 4.87] | 1.88  [-1.48 to 5.24] | 0.07  [-2.35 to 2.48] | -0.44  [-2.98 to 2.09] | 0.54  [-1.13 to 2.22] | 0.46  [-1.36 to 2.27] | 0.31  [-1.83 to 2.45] | | -0.22  [-2.48 to 2.03] |
| R5 (arb. unit) | 0.45  [-0.26 to 1.17] | 0.47  [-0.30 to 1.24] | -0.15  [-0.84 to 0.53] | -0.10  [-0.84 to 0.65] | 0.24  [-0.26 to 0.73] | 0.29  [-0.25 to 0.82] | 0.08  [-0.62 to 0.79] | | 0.00  [-0.78 to 0.78] |

^*^ Statistically significant differences between groups (P < 0.05) when analyzed using ANCOVA, adjusted for baseline values and compliance rate

**Abbreviations:** TEWL, transepidermal water loss; arb. unit, arbitrary unit

**Supplementary Table IV** Participants’ satisfaction with the product and skin outcomes after the 12-week intervention

| **Parameters** | **GEN^+^ Group (n=40)**  **(Mean ± SD)** | **PLA Group (n=40)**  **(Mean ± SD)** | | **P-value** |
| --- | --- | --- | --- | --- |
| **Product Satisfaction** | | | |  |
| 1) Overall satisfaction with the product over the past week | 8.30 ± 1.45 | | 8.08 ± 1.47 | 0.49 |
| 2) Overall satisfaction with the product as expected | 8.05 ± 1.68 | | 8.00 ± 1.45 | 0.89 |
| 3) Satisfaction with the product’s smell | 7.53 ± 2.11 | | 7.35 ± 1.78 | 0.69 |
| 4) Satisfaction with the product’s texture | 7.80 ± 2.23 | | 7.65 ± 1.35 | 0.72 |
| 5) Satisfaction with the product’s color | 8.38 ± 1.51 | | 8.23 ± 1.33 | 0.64 |
| 6) Satisfaction with the product’s toughness | 7.48 ± 2.11 | | 7.43 ± 1.63 | 0.91 |
| 7) Satisfaction with the product’s permeability through the skin | 7.70 ± 2.48 | | 7.90 ± 1.52 | 0.66 |
| **Skin Satisfaction** | | | |  |
| 1) Participant’s skin looks healthier | 8.20 ± 1.38 | | 7.88 ± 1.24 | 0.27 |
| 2) Participant’s skin looks stronger | 8.18 ± 1.45 | | 7.88 ± 1.32 | 0.34 |
| 3) Participant’s skin looks more moisturized | 8.50 ± 1.40 | | 8.13 ± 1.45 | 0.24 |
| 4) Participant’s skin looks smoother | 8.35 ± 1.42 | | 8.20 ± 1.32 | 0.63 |
| 5) Participant’s skin looks fresher | 8.28 ± 1.69 | | 8.08 ± 1.31 | 0.56 |
| 6) Participant’s skin looks brighter | 8.25 ± 1.55 | | 8.05 ± 1.30 | 0.53 |
| 7) Participant’s skin has better color consistency | 7.88 ± 1.68 | | 7.80 ± 1.57 | 0.84 |
| 8) Participant’s skin looks healthier after waking up | 8.18 ± 1.50 | | 7.98 ± 1.29 | 0.52 |
| 9) Participant’s skin looks cleaner | 8.30 ± 1.52 | | 8.10 ± 1.46 | 0.55 |
| 10) Participant’s pores appear improved | 7.88 ± 1.65 | | 7.78 ± 1.48 | 0.78 |
| 11) Participant’s redness appears reduced | 7.65 ± 1.83 | | 7.78 ± 1.48 | 0.74 |
